# A97 INTERNAL MEDICINE RESIDENT AND STAFF PERCEPTIONS OF GASTROENTEROLOGY ROTATION

**DOI:** 10.1093/jcag/gwad061.097

**Published:** 2024-02-14

**Authors:** N K Klemm, S Jayakumar

**Affiliations:** Gastroenterology, The University of British Columbia, Vancouver, BC, Canada; Gastroenterology, The University of British Columbia, Vancouver, BC, Canada

## Abstract

**Background:**

The choice of subspecialty by internal medicine residents is partially influenced by their experience with that service. Challenges exist between internal medicine and the high acuity, procedurally-heavy, gastroenterology (GI) service. Negative perceptions may limit the number of residents rotating through a gastroenterology elective, impacting knowledge and comfort managing common GI conditions.

**Aims:**

To identify misperceptions of the GI service at a tertiary hospital, and evaluate resident and new staff comfort in managing common GI conditions.

**Methods:**

Twenty-question survey sent to internal medicine residents during 2022-2023 and 13-question survey sent to staff that completed training from 2020-2022; both anonymous, and using Qualtrics software.

**Results:**

The survey was completed by 18% (30/166) of residents and 20% (13/65) of staff. Most staff (62%) practiced in a community setting. Both cohorts cited overnight cross-coverage consults during (56%) and negative word-of mouth (38%), as reasons they avoided a GI elective. Most participants reported a little (33%) or moderate (47%) amount of GI teaching on medicine service and were unaware of formal lectures (79%) during a GI rotation. Residents and staff were most comfortable managing pancreatitis (98%) and ALF (74%). Staff wished for more experience managing pancreatic and liver masses and outpatient IBD flares; however both groups (86%) were unaware of the ambulatory week during the GI rotation.

**Conclusions:**

Misperception and unawareness of a GI elective persist amongst internal medicine residents and has implications for new staff managing GI conditions. This study has led to action items that address these concerns.

Table 1: Resident & staff awareness, perceptions & knowledge of GI

**Funding Agencies:**

None

**Table:** 

	Resident	Staff
Reasons for not completing a GI rotation	n=30 (%)	n=9 (%)
I had adequate experience completing GI consults overnight as Cross-Coverage	15 (50)	7 (78)
GI service was too busy during the day	10 (33)	1 (11)
I heard from other residents that is was not a good rotation	10 (33)	5 (56)
I had not had a good experience with GI when on medicine service	7 (23)	2 (22)
Aware of ambulatory week	n=30 (%)	n=12 (%)
No	25 (83)	11 (92)
Aware of formal lectures	n=30 (%)	n=12 (%)
No	24 (80)	9 (75)
Amount of teaching on GI conditions during medicine service	n=30 (%)	n=13 (%)
None at all	2 (7)	0
A little	12 (40)	2 (15)
A moderate amount	10 (33)	10 (77)
A lot	6 (20)	0
A great deal	0	1 (8)
During my first year as staff, I felt residency prepared me to manage the following GI issues		n=13 (%)
ALF or ACLF		11 (84)
Liver Mass		5 (38)
IBD flare - inpatient		3 (23)
IBD flare - outpatient		0
Pancreatic mass		6 (46)
Pancreatitis & complications		11 (84)

